# Ovarian cancer metastasis to the breast 18 years after the initial diagnosis: A case report

**DOI:** 10.1097/MD.0000000000017577

**Published:** 2019-10-25

**Authors:** Zinan Wang, Dezhong Zhao, Ruobing Liu, Bin Zheng

**Affiliations:** Department of Breast, Yan’an Hospital of Kunming City, Kunming, Yunnan Province, China.

**Keywords:** breast neoplasms/secondary, ovarian neoplasms

## Abstract

**Rationale:**

Ovarian cancer often metastasizes, but it is unusual to transfer to the breast as an isolated mass. In particular, it is rare for patients to have breast metastases after 18 years of diagnosis of ovarian cancer. Therefore, accurate identification of ovarian cancer mammary gland metastasis can contribute to the treatment of the disease.

**Patient concerns:**

This case report shows that an 82-year-old woman was diagnosed with breast metastases from ovarian cancer diagnosed 18 years ago. The patient underwent total uterine attachment, omentum, pelvic lymphadenectomy, pelvic floor tumor reduction, and chemotherapy 18 years ago.

**Diagnosis:**

The pathological examination revealed metastatic adenocarcinoma. Immunohistochemical (IHC) staining results were negative for estrogen receptor (ER), progesterone receptor (PR), human epidermal receptor-2 (Her-2), and thyroid transcription factor-1 (TTF-1); and positive for cytokeratin (CK) 5/6, CK7, and CA125.

**Interventions:**

The patient underwent breast-conserving surgery and sentinel lymph node biopsy because of breast mass in November, 2018.

**Outcomes:**

Currently, she has been followed for more than 1 month without any signs of recurrence.

**Lessons:**

Breast metastatic tumors should be distinguished from primary breast tumors to avoid any unnecessary surgery. The correct diagnosis is very important: surgical treatment of patients with secondary breast cancer may be diagnostic and palliative.

## 1 Introduction

Mammary gland metastasis of ovarian cancer is very rare clinically. Sitzenfrey^[1]^ first reported a case of ovarian cancer metastasized to the breast in 1907. So far, only a few cases of ovarian cancer with breast metastasis have been reported worldwide.^[2]^ The treatment of patients with ovarian metastases to the breast is significantly different compared to patients with primary breast cancer. Ovarian cancer that metastasizes to the breast generally indicates rapid deterioration and death,^[2]^ so accurate diagnosis is important for subsequent treatment and prognosis. Here, we report a patient with mammary gland metastasis about 18 years after the initial diagnosis of ovarian cancer, and discussed the development of this rare disease.

## 2 Case report

The patient is an elderly woman, 82 years old this year. She went to the hospital for abdominal pain in May 2000. Color Doppler ultrasound showed moderate ascites and right ovarian accessory mass. Abdominal aspiration was taken as bloody osmotic fluid, and pathological smears found adenocarcinoma cells. She was then given preoperative neoadjuvant chemotherapy: intraperitoneal perfusion (cisplatin 120 mg) and systemic chemotherapy (cyclophosphamide 700 mg, pirarubicin 60 mg), every 3 weeks for 3 cycles.

On July 11, 2001, the patient underwent total uterine attachment, omentum, pelvic lymphadenectomy, and pelvic floor tumor reduction under general anesthesia. The postoperative examination was ovarian cancer. Adjuvant chemotherapy was performed after surgery: intraperitoneal perfusion (cisplatin 120 mg) and systemic chemotherapy (cyclophosphamide 700 mg, pirarubicin 60 mg), every 3 weeks for 3 cycles.

The patient was taken to the hospital because of the left breast mass on November 20, 2018. Physical examination showed that there was a hard mass in the left breast, which was not movable and painless, with a diameter of about 2 × 2 cm. The right breast and bilateral axillary fossa were normal. Molybdenum target showed a mass lesion in the lower quadrant of the left breast, which may be malignant. Doppler color Doppler showed that the low-echo nodules were found at 5 o’clock in the left breast. The shape was irregular, the edges were irregular, the right breast was not abnormal, and the bilateral lymph nodes were negative. Computed Tomography (CT) showed a small amount of effusion in the bilateral thoracic cavity; bilateral lungs were scattered in the nodules, with a maximum of 0. 7 × 0. 5 cm in the lateral segment of the right middle lobe.

**Figure 1:**
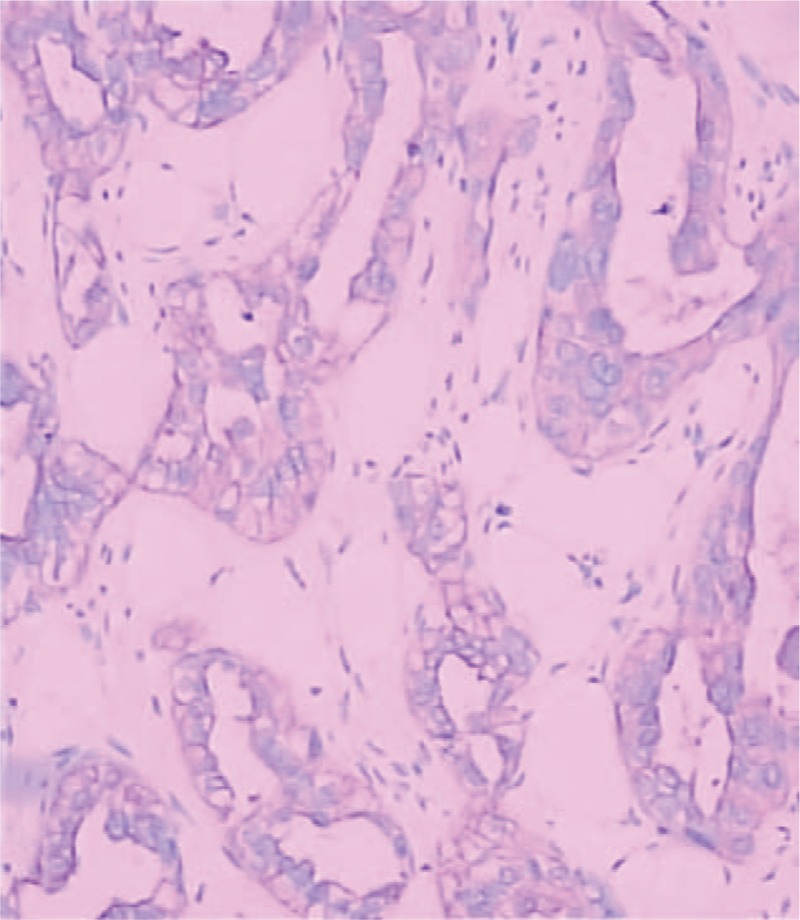
Pathological and immunohistological results. HE stain, ×100. HE = hematoxylin and eosin.

On November 21, 2018, she underwent general anesthesia for left breast cancer breast-conserving surgery and sentinel lymph node biopsy (SLNB). The pathological results were malignant, and no tumor metastasis was observed in peripheral tissues and sentinel lymph nodes. Immunohistochemical staining (Fig. 1) showed ER(−), PR(−), Her-2(−), Ki-67 (+40%), E-Cad (+), CK5/6(+), P53(−), S100 (+), CD34 (+), CA125 (+), CK7 (+), Napsin A (−), CK20 (individual weak +), CDX-2 (−), Villin (−), TTF-1 (−), WT-1 (−). Combined with immunohistochemistry and medical history, pathologists diagnosed ovarian cancer with mammary gland metastasis. At present, considering her age and other factors, she is recommended to conduct genetic testing, and feasible targeted therapy in the later stage. The patient has been followed up for >1 month and there are no signs of recurrence.

## 3 Method

This case was approved by the Institutional Review Board of Yan’an Hospital of Kunming City, Kunming, Yunnan, China. The patient signed the informed consent form and agreed to the publication of his information.

## 4 Discussion

Breast cancer is one of the most common primary malignancies in women, but tumors that metastasize to the breast are not common, accounting for only 0.5% to 1.3% of breast cancer cases.^[3]^ A study of 4051 breast cancer patients by Hadju and Urban found that the overall incidence of primary gynecologic tumor metastasis to breast cancer was 0.17%, and only 0.07% of metastatic disease was derived from primary ovarian tumors.^[4]^ Mammary metastases from primary ovarian tumors often lack specificity, it is morphologically indistinguishable from its original foci. The most common mode of metastasis of ovarian cancer is lymphatic metastasis.^[5]^ Lee et al speculate that there may be some dominant lymphatic pathways between other organs and the breast, such as bilateral lung cancer or gastric cancer, which increases the risk of metastatic cancer in the left breast through left supraclavicular lymph node metastasis, but this hypothesis has not been confirmed.^[6]^

In addition, mammary gland metastasis of primary ovarian tumors is generally diagnosed within an average of 2 years after initial diagnosis of ovarian cancer.^[7]^ The treatment and prognosis of breast metastasis from primary breast cancer and ovarian cancer are very different, so accurate diagnosis is necessary. Due to the rarity and unusual clinicopathological features of this tumor, it is difficult to make accurate clinical and histological diagnosis. Even if there is enough information and the patient is a known cancer patient, it is often difficult to distinguish whether it is primary or metastatic. In particular, the patient has been diagnosed with ovarian cancer for the past 18 years, and now it has been transferred to the breast, which makes us unable to make an accurate diagnosis before surgery. The prognosis of patients with ovarian cancer who have metastatic breast cancer is much worse than that of patients with primary breast cancer. Statistics on prognosis show that the one-year survival rate is about 40%.^[8]^

Mastectomy has little meaning in treating cancer that metastasizes to the breast, unless the tumor is very large or deep into the breast, which requires major surgery to achieve palliative resection.^[9]^ Therefore, accurate diagnosis of metastatic tumors in the breast will be properly treated, clinicians can eliminate unnecessary mastectomy. For patients who have received treatment for malignant tumors, metastatic breast cancer must be excluded by appropriate histopathological examination and comparison when there are multiple masses in breast tissue.

Breast cancer metastasis in patients with ovarian cancer is rare, and the disease is much worse than the prognosis of primary breast cancer,^[2]^ but as a clinician, we should be prepared to diagnose the disease.

## Author contributions

**Investigation:** Zinan Wang, Bin Zheng.

**Methodology:** Zinan Wang, Ruobing Liu, Bin Zheng.

**Supervision:** Dezhong Zhao, Ruobing Liu, Bin Zheng.

**Writing – original draft:** Zinan Wang.

**Writing – review & editing:** Dezhong Zhao, Bin Zheng.
